# A Case Report on an Underappreciated Cause of Heart Failure: Chronic Viral Myocarditis

**DOI:** 10.7759/cureus.27253

**Published:** 2022-07-25

**Authors:** Sandra K Rabat, Usman Manzoor, Naeem Ijaz, Mark M Aloysius

**Affiliations:** 1 Internal Medicine, A.T. Still University School of Osteopathic Medicine, Mesa, USA; 2 Internal Medicine, The Wright Center for Graduate Medical Education, Scranton, USA

**Keywords:** coxsackie b, viral myocarditis, viral-induced myocarditis, heart failure with reduced ejection fraction, dilated cardiomyopathy

## Abstract

A 64-year-old male with a history of congestive heart failure (CHF), coronary artery disease status post two stents in 2014, hypertension, and chronic kidney disease (CKD) stage III, was admitted for acute exacerbation of CHF. Treatment started with blood pressure control and high-dose diuretics. While the patient’s volume status improved, his clinical status declined, and he required a dobutamine infusion. Cardiac catheterization revealed nonischemic cardiomyopathy. He was ultimately found to have myocarditis secondary to chronic Coxsackie B infection. A comprehensive investigation ruled out other potential etiologies. This case highlights how viruses continue to be an underappreciated cause of heart failure. Infectious agents should not be underestimated as several types of viral infections carry substantial cardiovascular risks, potentially leading to significant deterioration in decompensated patients.

## Introduction

The diagnosis of myocarditis continues to be a challenge for clinicians as characterization of this disease has been hindered by its diverse etiologies and clinical presentations [1]. Among the culprits of vast etiologies are viral infections, which can discreetly but occasionally result in symptomatic heart failure, requiring inotropic support. Viral myocarditis presents with non-specific symptoms, including chest pain, dyspnea, and palpitations, mimicking more common disorders such as coronary artery disease [1]. We present a 64-year-old patient who presented initially with shortness of breath. He was admitted for acute exacerbation of congestive heart failure (CHF) with an ejection fraction of 10%, requiring inotropic treatment with dobutamine and milrinone. Cardiac catheterization revealed nonischemic cardiomyopathy. He was ultimately found to have chronic Coxsackie B myocarditis. This case underlines the importance of maintaining a broad differential when approaching cases of heart failure.

## Case presentation

A 64-year-old male with a past medical history of CHF, coronary artery disease status post left anterior descending artery and right coronary artery stenting in 2014, hypertension, and chronic kidney disease (CKD) stage III presented to our hospital for progressive shortness of breath for two weeks. The patient reported shortness of breath with walking for about 10-15 feet, 3-pillow orthopnea, and worsening lower extremity edema lasting all day. He denied any events or triggers that could have exacerbated his shortness of breath. He did not require home oxygen therapy or inhalers at home. The patient had poor compliance with his home medications, including carvedilol, lisinopril, furosemide, atorvastatin, and clopidogrel, and stated he took them irregularly, if at all. A review of systems was remarkable for a feeling of rapid heartbeat but otherwise negative. He had a significant family history of premature coronary artery disease and stated his grandfather had sudden cardiac death at age 49. The patient denied any illicit drug use, smoking, or alcohol use. He had significant social determinants of health, including experiencing homelessness and a lack of social support from family. 

**Figure 1:**
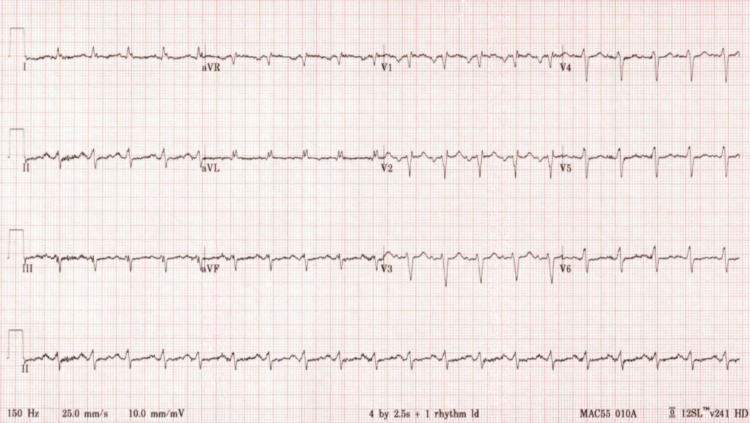
EKG showing prolonged QTC interval, left-axis deviation, and non-specific ST-T changes.

**Figure 2:**
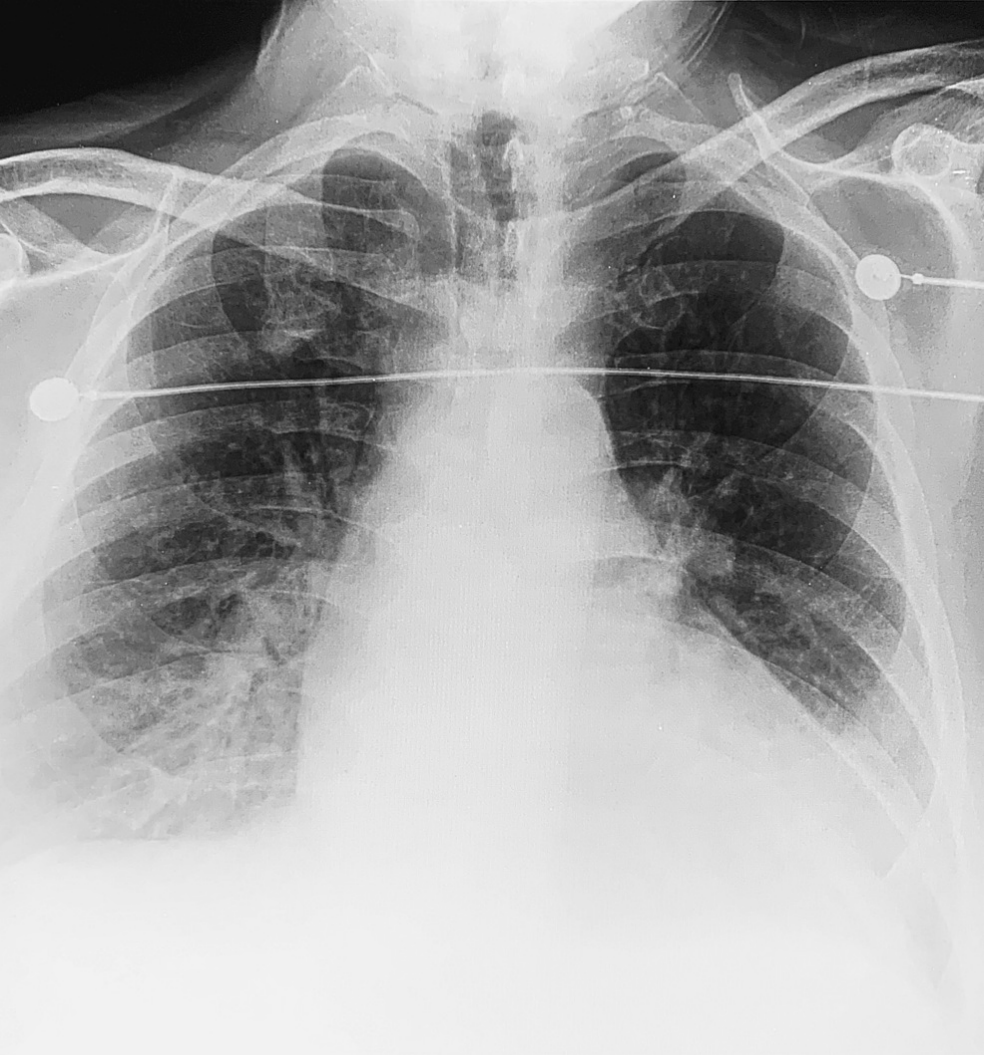
Chest X-ray demonstrating bilateral pulmonary edema and cardiomegaly supportive of CHF. CHF: Congestive heart failure.

On initial presentation, the patient was found to be in a hypertensive emergency with a blood pressure of 220/110 mmHg. He was saturating at 84% oxygen on room air. Labs were significant for creatinine of 2.4 mg/dL, which was elevated from his baseline of 1.7 mg/dL. His troponins and brain natriuretic peptide (BNP) were 12,333 pg/ml and 1,431 pg/ml, respectively. We recognize the challenge in interpreting cardiac troponin levels and BNP in the setting of CKD. However, the magnitude of elevation of the troponins and BNP was very concerning for another process within the myocardium rather than being a false-positive elevation from CKD alone. EKG showed prolonged QTC interval, left-axis deviation, non-specific ST-T changes, and absence of ST-segment elevations (Figure 1). Chest X-ray revealed cardiomegaly with pulmonary edema (Figure 2). The elevated troponin levels and EKG results ruled out ST-segment elevation myocardial infarction (STEMI) and were likely consistent with non-ST-segment elevation myocardial infarction (NSTEMI). The elevated BNP levels and chest X-rays were strongly supportive of CHF. He was treated with one dose of clonidine, nasal cannula oxygen, and heparin drip in the ED. He was subsequently started on hydralazine as needed for systolic blood pressure of more than 160 mmHg. Aggressive diuresis with IV furosemide and accelerated cardiac work-up was initiated as he was determined to have acute exacerbation of CHF.

**Figure 3:**
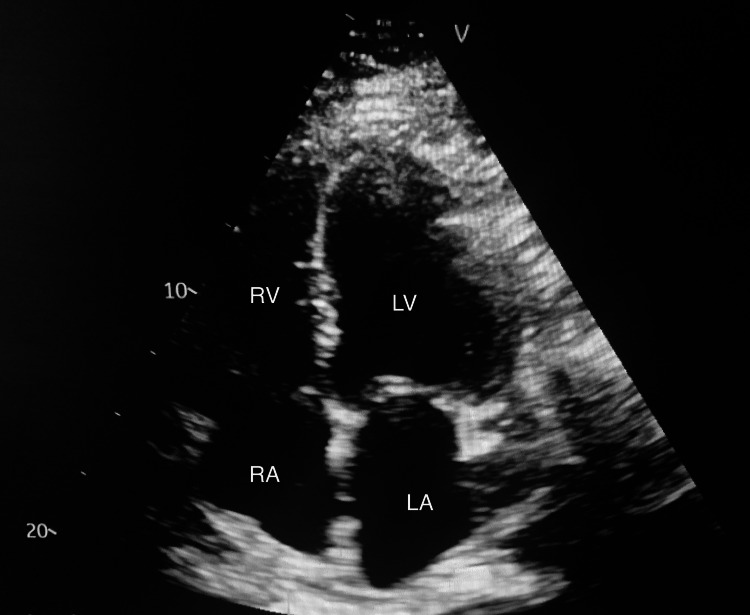
TTE (apical 4 chamber view) showing dilated cardiac chambers including mild dilation of the left ventricle (LV), right atrium (RA), and right ventricle (RV), and moderate dilation of the left atrium (LA). The ejection fraction was estimated at 10% with severely impaired left ventricular systolic function, impaired right ventricular systolic function, and grade three diastolic dysfunction. TTE: Transthoracic echocardiogram.

**Figure 4:**
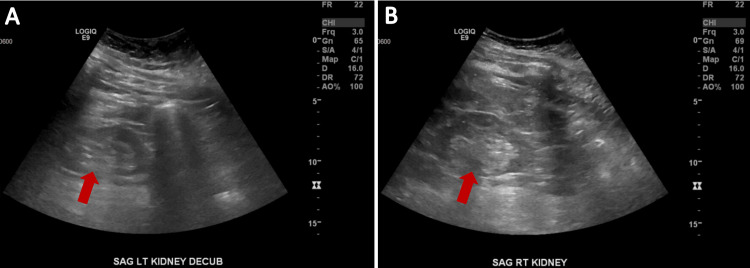
Abdominal ultrasound demonstrating bilateral renal atrophy with diffusely increased echogenicity bilaterally, consistent with CKD. CKD: Chronic kidney disease.

Work-up for pulmonary embolus was unremarkable, as evidenced by negative venous duplex and V/Q scan. Transthoracic echocardiogram (TTE) revealed significant findings, including an estimated ejection fraction of 10% with moderate mitral regurgitation and moderate tricuspid regurgitation, a dilated right ventricle with severely impaired systolic function, and grade three diastolic dysfunction with restrictive filling (Figure 3). Of note, his last echocardiogram completed a little less than two years ago showed an estimated ejection fraction of 60% with preserved left ventricular systolic function. The patient was given a LifeVest (ZOLL Medical Corporation, Chelmsford, Massachusetts), a portable external cardiac defibrillator system, due to his severe left ventricular dysfunction and dilated left ventricle. Abdominal ultrasound showed bilateral renal atrophy with diffusely increased echogenicity bilaterally, indicative of CKD (Figure 4). The patient was in volume overload, and diuresis was continued with close monitoring of creatinine levels. After interval improvement of renal functions, the patient subsequently underwent a cardiac catheterization, which revealed nonobstructive coronary artery disease and severe pulmonary hypertension. Right heart hemodynamics revealed mean pulmonary capillary wedge pressure (PCWP) of 40 mmHg, mean pulmonary artery (PA) pressure of 60 mmHg, and mean right atrial (RA) pressure of 32 mmHg. Nonischemic cardiomyopathy clearly was not a cause of his acute decompensation.

After the procedure, diuretics were withheld, and he was started on dobutamine infusion at 5 mcg/kg/min, which was titrated to a mean arterial pressure of at least 65 mmHg. The patient was then started on isosorbide mononitrate and hydralazine and continued on carvedilol. He was resumed on diuretics with torsemide and was not a candidate for angiotensin-converting enzyme (ACE) inhibitor or angiotensin receptor neprilysin inhibitor (ARNI) due to his acute kidney injury superimposed on CKD stage III with a glomerular filtration rate (GFR) of less than 30 ml/min/1.73 m2. We attempted to wean the patient off dobutamine, but his kidney function worsened to creatinine of 2.7 mg/dL. This indicated that inotropic support was most likely needed in his case. Once renal function improved, milrinone infusion was initiated and followed closely. The indication for milrinone was for decompensated heart failure with low cardiac output and evidence of end-organ hypoperfusion. The goal was to combine milrinone infusion with standard heart failure therapy, including a beta-blocker, as tolerated. The benefit of using milrinone over dobutamine in this patient’s case is that milrinone, a phosphodiesterase inhibitor, will not antagonize a beta-blocker like dobutamine. Dobutamine’s action is partly on beta-1 and beta-2 adrenergic receptors; thus, concomitant beta-blocker therapy can be expected to blunt the patient’s hemodynamic response to dobutamine. The patient was to undergo a cardiac MRI and possible endomyocardial biopsy. However, the viral panel was positive for Coxsackie B viral antibody immunoglobulin G (IgG), indicating chronic viral infection. 

## Discussion

Cardiomyopathy is an anatomic and pathologic diagnosis associated with muscle or electrical dysfunction of the heart [2]. The American Heart Association (AHA) defines cardiomyopathy as a heterogeneous group of diseases of the myocardium, usually with inappropriate ventricular hypertrophy or dilatation [3]. There are four major types of cardiomyopathy, including dilated cardiomyopathy, hypertrophic cardiomyopathy, restrictive cardiomyopathy, and arrhythmogenic right ventricular cardiomyopathy [3]. Dilated cardiomyopathy in adults is most commonly caused by ischemic cardiomyopathy and hypertension; however, viral myocarditis, valvular disease, and genetic predisposition also play a role [2]. In fact, viral myocarditis is an underdiagnosed cause of acute heart failure and chronic dilated cardiomyopathy. Another commonly missed trigger of acute heart failure in patients with dilated cardiomyopathy is iron deficiency anemia [4]. 

The incidence rate of viral myocarditis is 10-22 per 100,000 individuals [1]. However, the exact incidence of myocarditis is difficult to ascertain, with many undiagnosed and unreported cases [5]. Moreover, viral myocarditis is often overlooked due to its varied presentation ranging from non-specific symptoms of shortness of breath to more aggressive symptoms that mimic acute coronary syndrome. Indeed, one study screened 3055 patients with suspected acute or chronic myocarditis and revealed that 72% had dyspnea, 32% had chest pain, and 18% had arrhythmias [6]. A large spectrum of viruses has been implicated as causes of myocarditis, including Coxsackievirus B, parvovirus B19, adenovirus, Epstein-Barr virus, HIV, and, more recently, COVID-19 [6-7]. Coxsackie B virus is one of the most common causes of viral myocarditis and is responsible for 10-20% of all myocarditis and dilated cardiomyopathy cases [8].

Myocarditis typically results from cardiotropic viral infection followed by active inflammatory destruction of the myocardium [9]. Furthermore, there are three possibilities that may result after the initial acute symptoms of viral myocarditis, including the following: the virus may be completely cleared, resulting in full recovery, the viral infection may persist, or the viral infection could lead to a persistent auto-immune-mediated inflammatory process with long-term symptoms of heart failure [10]. Viral persistence in the myocardium is associated with progressive deterioration of left ventricular ejection fraction (LVEF) [11]. This was likely the case with our patient’s new drop in ejection fraction from an LVEF of 60% to an LVEF of 10% over less than two years. Endomyocardial biopsy (EMB) is the gold standard method for diagnosing acute or chronic inflammatory heart disease [11]. However, EMB is used infrequently because of perceived risks and the lack of a widely accepted and sensitive histologic standard [6]. Liquid biopsy by monitoring circulating biomarkers, including microRNAs (miRNAs), has also been shown to have the potential to complement EMBs and provide the excellent diagnostic capability. A recent study revealed the ability to discriminate between patients with viral myocarditis, inflammatory cardiomyopathy, and healthy donors with a specificity of over 95% based on expression levels of miRNAs [11]. However, further studies would be needed to elevate the routine use of miRNA-panel in addition to further guidelines to help optimize the management of this disease. Based on current guidelines, patients are treated symptomatically with optimal heart failure medications [12].

## Conclusions

The current COVID-19 pandemic has brought to light a special global sensitivity to viral infections. Viral myocarditis still remains an underdiagnosed cause of acute heart failure and chronic dilated cardiomyopathy. The diagnosis, prognosis, and treatment can be highly unpredictable and challenging. Diagnostic advances in cardiac MRI, molecular detection of viruses by EMB, and liquid biopsies have improved the ability to diagnose and understand the pathophysiological mechanisms of this disease. Despite recent research developments, viral myocarditis and its sequelae leading to heart failure are still not fully understood and constitute a significant public health issue worldwide. This case emphasizes the importance of maintaining a wide differential when approaching cases of heart failure. It is imperative to consider that even chronic Coxsackie B viral infection can cause an acute presentation of heart failure.
